# The metastasis suppressor SOX11 is an independent prognostic factor for improved survival in gastric cancer

**DOI:** 10.3892/ijo.2014.2328

**Published:** 2014-03-06

**Authors:** YING QU, CHENFEI ZHOU, JIANIAN ZHANG, QU CAI, JIANFANG LI, TAO DU, ZHENGGANG ZHU, XIAOJIANG CUI, BINGYA LIU

**Affiliations:** 1 Shanghai Key Laboratory of Gastric Neoplasms, Shanghai Institute of Digestive Surgery, Department of Surgery, Ruijin Hospital, Shanghai Jiao Tong University School of Medicine, Shanghai 200025, P.R. China;; 2 Department of Surgery, Department of Obstetrics and Gynecology, Women’s Cancer Institute, Samuel Oschin Comprehensive Cancer Institute, Cedars-Sinai Medical Center, Los Angeles, CA 90048, USA

**Keywords:** SOX11, gastric cancer, prognostic factor, metastasis

## Abstract

SOX11 is involved in gastrulation and in malignant diseases. The aim of this study was to investigate the role of SOX11 in gastric cancer and its expression pattern and clinical significance. SOX11 overexpression cell model was used to examine *in vitro* and *in vivo* the role of SOX11 in cell growth and metastasis. Cell cycle analysis and Annexin V/PI double staining were used to investigate the effect of SOX11 on cell cycle progression and apoptosis. The expression of SOX11 in human gastric cancer was examined by immunohistochemistry. The correlation of SOX11 expression with clinicopathological characteristics and survival of patients was analyzed by Pearson’s χ^2^ and Kaplan-Meier analyses, respectively. Cox’s proportional hazard model was employed in multivariate analysis. SOX11 overexpression did not inhibit cell growth but strongly suppressed cell migration/invasion *in vitro* and *in vivo*. We found a significant correlation between high SOX11 protein levels and Lauren’s classification (intestinal type), differentiation status (high and medium), and early TNM stage. SOX11 is an independent prognostic factor for improved survival in gastric cancer patients. SOX11 was a potential tumor-suppressor and an independent positive prognostic factor in gastric cancer patients with less advanced clinicopathological features.

## Introduction

Although its incidence rate has steadily declined in recent decades, gastric cancer remains a global health problem. Gastric carcinoma is the fourth most common malignancy in the world, with an estimated 989,000 new cases and 738,000 deaths reported in 2008. The depth of invasion and the presence of lymph node metastases are considered to be the most important prognostic factors in gastric cancer (1,2). However, investigating molecular biomarkers not only can provide indications for clinic prognosis, but also can identify potential targets for clinical therapy.

SOX [sex-determining region Y (Sry) box containing] factors are a family of structurally-related transcription factors with potent effects on cellular phenotypes. SOX11 belongs to group C of mammalian SOX proteins. It has two functional domains: a Sry-related HMG box (SOX) DNA-binding domain, located in the N-terminal half of the protein, and a transactivation domain (TAD) located at the C-terminus (3). The HMG box domain alone fulfills the functions of DNA binding, DNA bending, protein interactions and nuclear import or export. The SOX HMG box domain contains two nuclear localization domains that are independent of each other and that have been highly conserved in all SOX proteins (4). The Sox EHMG box domain also features a nuclear export signal (5,6).

To date, the main function of SOX11 in non-malignant tissues is in neural development and organogenesis (7) during fetal development. SOX11 is present during gastrulation and early postgastrulation development throughout the embryo (8). Later during development, SOX11 is prominently expressed in the developing nervous system in both glial and neuronal lineages and at many sites throughout the embryo where epithelial-mesenchymal interactions occur (8). At sites of such epithelial-mesenchymal interactions, SOX11 can be found in the mesenchymal or epithelial compartment, and it has been postulated to be involved in inductive remodeling (8). SOX11-depletion can cause death at birth and other severe developmental defects (9). Among which, abdominal wall closure defects, asplenia, and stomach hypoplasia are major phenotypes of SOX11-deficiency in gut development (9).

SOX11 was firstly reported in hematopoietic malignancies such as lymphoma (10). SOX11 is considered as a diagnostic and prognostic antigen in B cell lymphomas (11,12) and has been demonstrated to have tumor suppressor functions (10). Recently, SOX11 has been studied in other solid tumors. The presence of SOX11 is associated with improved recurrence-free survival (RFS) in ovarian cancer (13). However, the studies on the expression and significance of SOX11 in malignant tumors other than lymphoma are very limited.

In this study, we examined the role of SOX11 in gastric cancer using *in vitro* and *in vivo* models. Immunohistochemistry was used to investigate the expression pattern of SOX11. The correlations of SOX11 with patient clinicopathological features were analyzed and the prognostic significance of SOX11 was evaluated.

## Materials and methods

### Patients and specimens

Gastric cancer tissues, confirmed by pathological diagnosis, were obtained from 151 patients who underwent radical resection for gastric cancer between 2006 and 2008 at the Department of Surgery, Ruijin Hospital, Shanghai, China. The corresponding non-tumor gastric tissue was obtained at least 6 cm from the tumor. All tissue samples were formalin-fixed and paraffin-embedded. Clinicopathological and survival data of all patients were collected. TNM staging was classified based on the criteria of American Joint Committee on Cancer (AJCC, 7th edition) for gastric cancer. The mean age of the patients at initial surgery was 65 years (range, 34–84 years); 104 men and 47 women were included in this study. The mean duration of follow-up was 39 months (range, 1–73 months). The AJCC tumor stage distribution and vital status of the patients are shown in Table I. The study was approved by the Shanghai Jiao Tong University Medical School institutional review board.

### Immunohistochemistry staining

Immunohistochemistry (IHC) staining was performed using a highly sensitive streptavidin-biotin-peroxidase detection system with gastric cancer tissue microarrays. Mouse monoclonal anti-SOX11 at a dilution of 1:100 (Cell Marque, CA, USA) was used as previously reported (14). Mayer’s haematoxylin was used for counterstain. The slides were evaluated by a single board-certified pathologist (RRT) who remained blinded to the clinical data using standard light microscopy.

### Immunohistochemistry assessment

Expression status of SOX11 was determined by using a semi-quantitative scoring system based on percentage and staining intensity of positive cells. The percentage of positive cells was divided into five grades (percentage scores): <10% (0), 10–25% (1), 25–50% (2), 50–75% (3) and >75% (4). The staining intensity was divided into four grades (intensity scores): no staining (0), weak staining (1), moderate staining (2) and strong staining (3). SOX11 staining status was determined by the following formula: overall score = percentage score x intensity score. The overall score ≤3 was defined as negative, and >3 was defined as positive.

### Gastric cancer cell lines and cell culture

Gastric cancer cell lines AGS, N87, MKN45, MKN28, SGC-7901, KATOIII, BGC823 and Hs746T were preserved in the institute. The cells were grown in RPMI-1640 medium containing 10% fetal bovine serum (FBS), penicillin and streptomycin (Gibco BRL, Gaithersburgh, MD, USA). Plasmid containing Myc-DDK-tagged ORF clone of *Homo sapiens* SOX11 was purchased from Origene (Origene Technologies, MD, USA). Cell proliferation was assessed by the 3-(4,5-dimethylthiazol-2-yl)-2,5-diphenyltetrazolium (MTT) (Sigma, St. Louis, MO, USA) assay. Soft agar colony formation assay was performed by using 0.3% agar in complete medium with cells as the feeder layer and 0.6% agar in complete medium as the bottom layer. 3D Matrigel culture was performed using Matrigel matrix (BD Biosciences, San Jose, CA, USA).

### Quantitative real-time PCR (qRT-PCR)

Total RNA was isolated from cultured cells using the RNeasy mini kit (Qiagen) and cDNA was synthesized with oligo(dT) primers by using a SuperScript first-strand cDNA synthesis kit (Invitrogen) according to the manufacturer’s protocols. Gene expression was assessed by qRT-PCR using an Applied Biosystems 7500 Fast Sequence Detection System (Life Technologies Corp., CA, USA). The PCR reaction mixture consisted of QuantiTect SYBR Green PCR master mix (2X QuantiTect SYBR Green kit, contains HotStart Taq^®^ DNA polymerase, QuantiTect SYGB Green PCR buffer, dNTP mix, SYGB I, Rox passive reference dye and 5 mM MgCl_2_) (Qiagen), 0.5 *μ*mol/l of each primer and cDNA. The transcript of the housekeeping gene, glyceraldehyde-3-phosphate dehydrogenase (GAPDH) gene was used as endogenous control to normalize expression data. Primers used for qRT-PCR analysis of SOX11 expression are 5′-GGTGGATAAGGATTTGGATTCG-3′ (forward) and 5′-GCTCCGGCGTGCAGTAG T-3′ (reverse). Primers used for analysis of GAPDH are 5′-TTGGCATCGTTGAGGGTCT-3′ (forward) and 5′-CAGTGGGAACACGGAAAGC-3′ (reverse). The comparative Ct (threshold cycle) method was used to calculate the relative changes in gene expression.

### Western blotting

Whole cell lysates were harvested using RIPA cell lysis buffer supplemented with a protease inhibitor cocktail (Sigma). The nuclear and cytosol extracts were isolated using the nuclear extract kit (Active Motif, Carlsbad, CA, USA) according to the manufacturer’s instructions. Rabbit monoclonal antibody against SOX11 (Epitomics) were used at 1:1,000 dilutions. The signals were visualized using Li-COR Odyssey-Sa model 9260 (Li-COR Corp., USA), and images were taken and managed using Odyssey Sa Infrared Image System (Li-COR Corp.).

### Flow cytometry

For cell cycle analysis, single cells were fixed in 70% ice-cold ethanol at 4˚C overnight, washed with PBS, incubated with 100 *μ*g/ml RNase at 37˚C for 20 min. After staining with propidium iodide (50 *μ*g/ml), the cells were subjected to fluorescence activated cell sorting (FACS). For apoptosis analysis, Annexin V/PI (BD Biosciences) double staining was used and followed by FACS analysis. FACSCalibur was used in flow cytometry and the data were analyzed by Cell Quest software (BD Biosciences).

### Immunofluorescence staining

Cells plated on glass coverslips were washed with PBS and fixed in 4% paraformaldehyde for 15 min at room temperature. Monolayers were washed with PBS, blocked with 5% BSA for 30 min and incubated with the primary antibody diluted in blocking solution for 2 h at room temperature. After washing, the cover slips were incubated with cyanine-3- and/or cyanine-2-conjugated secondary antibodies for 30 min, washed three times in PBS and mounted. DAPI (4′,6-diamidino-2-phenylindole) staining [1:5,000 (vol/vol) of a 5 mg/ml stock] was used to visualize DNA. Immunofluorescence staining was visualized using Olympus BX50 microscope (Olympus Opticol Co., Japan), images were taken using Nikon Digital Sight DS-U2 (Nikon, Japan) and NIS elements F3.0 software was used (Nikon).

### Cell migration and invasion assays

Cell migration was analyzed by a transwell chamber assay. Cell invasion assays were performed using BD BioCoat^™^ Matrigel^™^ Invasion Chambers. FBS (10%) was used as the chemoattractant. Cells on the lower surface of the insert were fixed and stained followed by counting under a light microscope. Cells were visualized using Olympus BX50 microscope (Olympus Opticol Co.), images were taken using Nikon Digital Sight DS-U2 (Nikon, Japan) and NIS elements F3.0 software was used (Nikon).

### In vivo tumorigenesis and metastasis

Male BALB/c nu/nu nude mice (Institute of Zoology Chinese Academy of Sciences, Shanghai, China), were housed at a specific pathogen-free environment in the Animal Laboratory Unit, School of Medicine, Shanghai Jiao Tong University, China. Mice received humane care and the study protocols comply with the Institution’s guideline and animal research laws. Cells (1×10^6^) were subcutaneously injected into 4-week-old male BALB/c mice. The growth of primary tumors was monitored every 3 days by measuring tumor diameters. Tumor length (L) and width (W) were measured and tumor volume was calculated by the equation: volume = (W^2^ × L)/2 (15). Mice were sacrificed 28 days after injection under anesthesia. Eight mice were used in each group.

To produce peritoneal spreading experimental metastasis, 2×10^6^ cells were injected into 5-week-old male BALB/c nude mice intraperitoneally. After 6 weeks, the mice were sacrificed under anesthesia. Ten mice were used in each group. Macrometastatics were visualized and counted.

### Statistical analyses

The differences in clinicopathological features between different groups were determined using Pearson’s χ^2^ test. The survival curves of each group were estimated by Kaplan-Meier survival analyses, and the curves were compared using log-rank tests. In multivariate analysis, a Cox’s proportional hazard model was applied to determine whether a factor was an independent predictor of survival. The statistical analyses were performed using SPSS 13.0 software (SPSS Inc., Chicago, IL, USA). Values are presented as the mean ± standard deviation (SD) of samples measured in triplicate. Each experiment was repeated three times, unless otherwise indicated. The significance of differences between experimental groups was analyzed using the Student’s t test and two-tailed distribution.

## Results

### Database analysis of SOX11 mRNA expression in human gastric cancer

**Figure 1.**
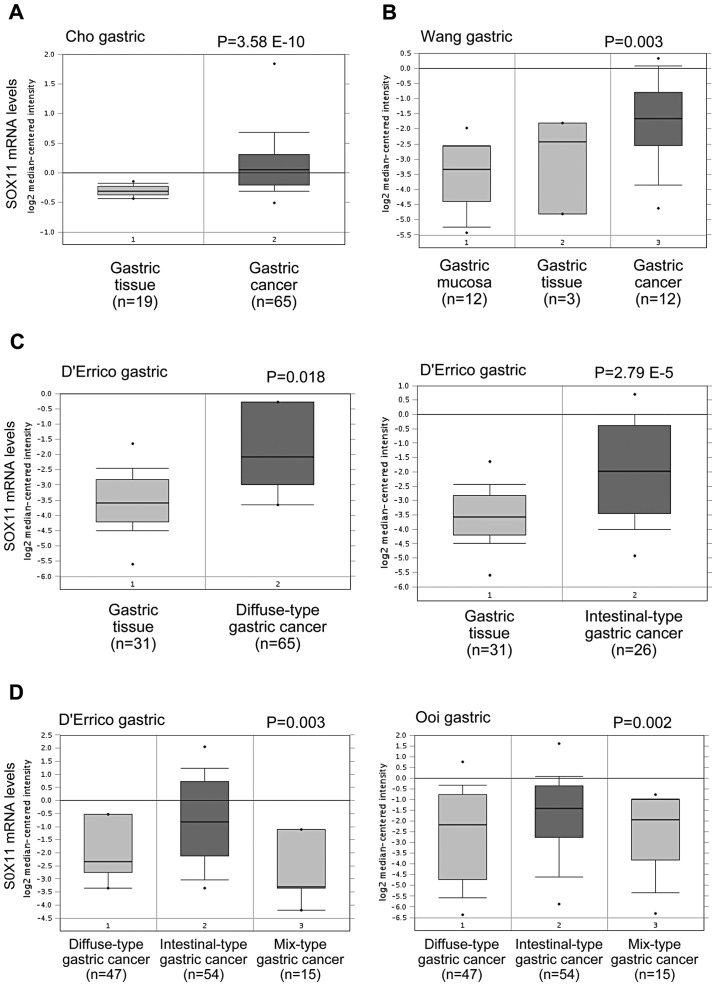
Expression of SOX11 mRNA levels in human gastric cancers using the Oncomine database. Analysis of SOX11 mRNA levels in human gastric cancer tissues compared with gastric normal tissues. The mRNA levels of SOX11 in human gastric cancers and normal gastric tissues in Cho (A), Wang (B) and D’Errico (C) datasets from the Oncomine database are shown. (D) Analysis of SOX11 mRNA levels in gastric cancer tissues with Lauren’s classification. D’Errico and Ooi datasets from the Oncomine database are shown.

We first investigated the SOX11 mRNA levels in human gastric cancer using the datasets from the publicly available Oncomine database (www.oncomine.org). SOX11 mRNA was significantly elevated in human gastric cancer tissues compared with normal tissues in the Cho *et al* (16), Wang *et al* (17) and D’Errico *et al* (18) datasets from the Oncomine database (Fig. 1A–C). Furthermore, SOX11 mRNA levels were significantly higher in gastric cancer of intestinal-type than other types in Ooi *et al* (19) and D’Errico *et al* (18) datasets (Fig. 1D). These data suggest that SOX11 mRNA level is upregulated in human gastric cancer.

### Expression of SOX11 in gastric cancer cell lines

**Figure 2.**
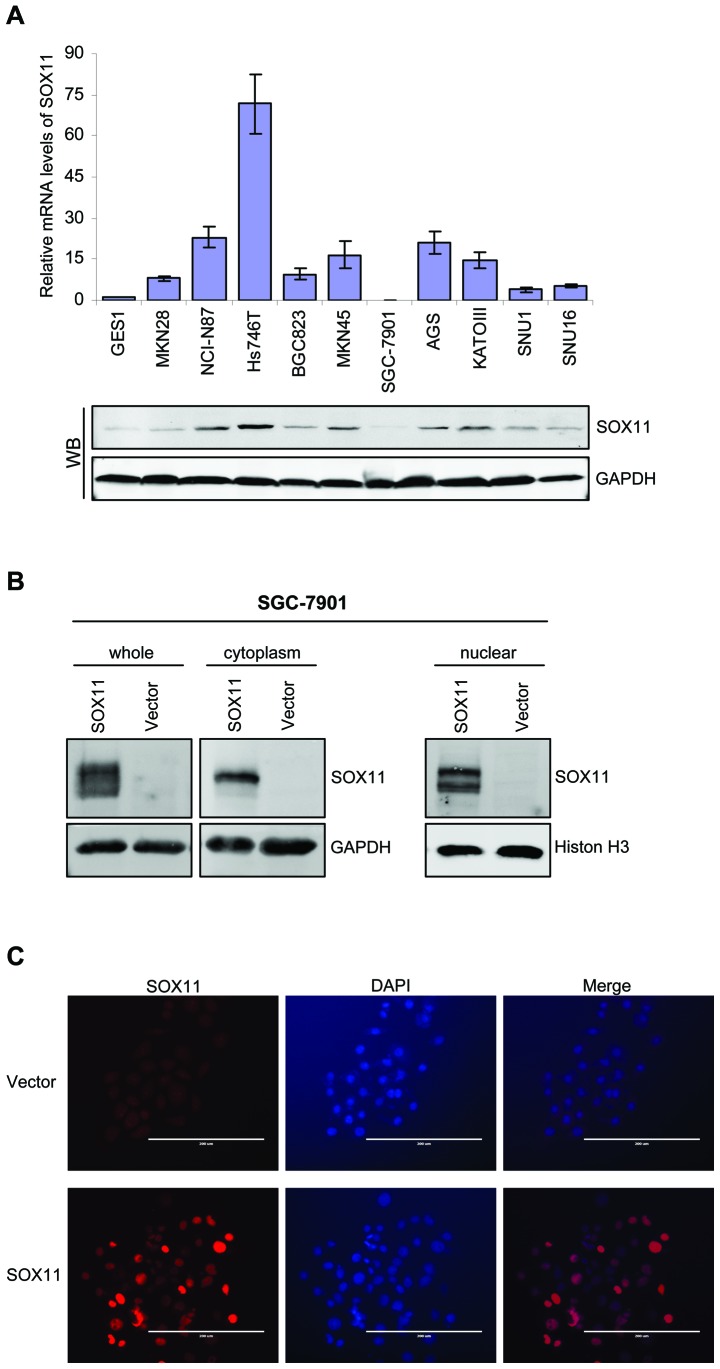
Expression of SOX11 in gastric cancer cell lines. (A) SOX11 mRNA and protein levels in ten gastric cancer cell lines and immortalized normal gastric epithelial cell line GES-1. Data represent mean ± SD of three independent experiments in column plot. (B) Immunoblotting analysis of SOX11 levels in SOX11-overexpressing and vector control cells using whole cell, cytoplasm and nuclear fraction. GAPDH was used as a loading control for whole and cytoplasm extracts. Histone H3 was used as a loading for nuclear extract. (C) Immunofluorescence staining of SOX11 in SOX11-overexpressing and vector-control cells. Bar, 200 *μ*m.

We then analyzed the SOX11 expression in human gastric cancer cell lines and the immortalized normal gastric epithelial cell line GES-1. We performed qRT-PCR and immunoblotting to analyze the SOX11 mRNA and protein levels in gastric cancer cell lines and GES-1. Consistent with database analysis, we showed that SOX11 mRNA and protein levels were highly expressed in all gastric cancer cell lines compared to GES-1. Notably, five gastric cancer cell lines (NCI-N87, Hs746T, AGS, KATO-III) showed higher and five cell lines (MKN28, BGC823, SGC-7901, SNU1, SNU16) showed lower SOX11 protein levels (Fig. 2A). These data demonstrate that SOX11 is highly expressed in some gastric cancer cell lines.

### Ectopic overexpression of SOX11 in gastric cancer cells does not affect growth in vitro and in vivo

**Figure 3.**
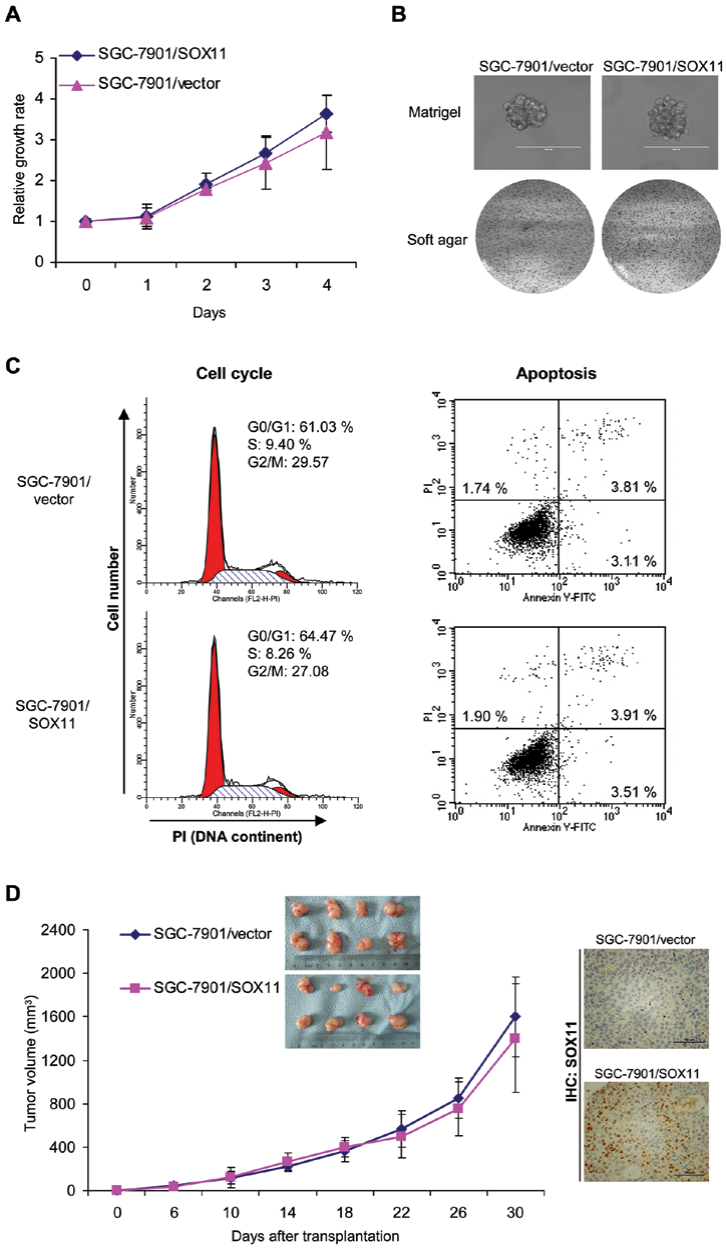
SOX11 overexpression did not affect gastric cancer cell growth *in vitro* and *in vivo*. Overexpression of SOX11 in SGC-7901 gastric cancer cells did not inhibit growth under monolayer (A) or in soft agar/Matrigel culture (B). (C) Cell cycle and apoptosis analysis using SOX11-overexpressing and vector-only SGC-7901 cells. (D) Growth curves of tumors after injection of control and SOX11-overexpressing SGC-7901 cells in nude mice. Data are shown as the mean ± SD (n=8). Representative images show tumors at the day of scarifice. Immunohistochemistry staining was used to examine the SOX11 expression in tumors from SOX11-overexpressing and vector-only SGC-7901 cells.

We generated SOX11 overexpression models using SGC-7901 (Fig. 2B) and MKN45 cell line (data not shown), which did not express or expressed weak levels of SOX11. Immunofluorescence staining also revealed strong nuclear signaling in SOX11-overexpressing SGC-7901 cells (Fig. 2C). To investigate the role of SOX11 in the growth of gastric cancer cells, we first examined the cell proliferation in monolayer culture. As shown in Fig. 3A, SOX11 overexpression did not affect cell growth in monolayer culture. We then performed soft agar and Matrigel 3D culture. We found that SOX11 overexpression did not suppress the growth of SGC-7901 (Fig. 3A) and MKN45 (data not shown) gastric cancer cells in soft agar and Matrigel (Fig. 3B). We then analyzed cell cycle division and apoptosis by flow cytometry. Cell cycle distribution and apoptotic rate did not show significant difference between vector-control and SOX11-overexpressing cells (Fig. 3C).

In order to examine the effects of SOX11 on the *in vivo* growth of gastric cancer cells, we employed two experimental models. Control and SOX11-overexpressing SGC-7901 cells were injected orthotopically into nude mice and tumor growth was examined. Mice injected with vector-control (SGC-7901/vector) and SOX11-overexpressing (SGC-7901/SOX11) cells formed similar size tumors within 37 days (Fig. 3D, left). The nuclear SOX11 was observed in tumors formed by SOX11-overexpressing cells but not in that formed by vector-control cells (Fig. 3D, right). These data suggested that SOX11 overexpression did not affect gastric cancer cell growth.

### Ectopic overexpression of SOX11 in gastric cancer cells inhibits invasion in vitro and in vivo

**Figure 4.**
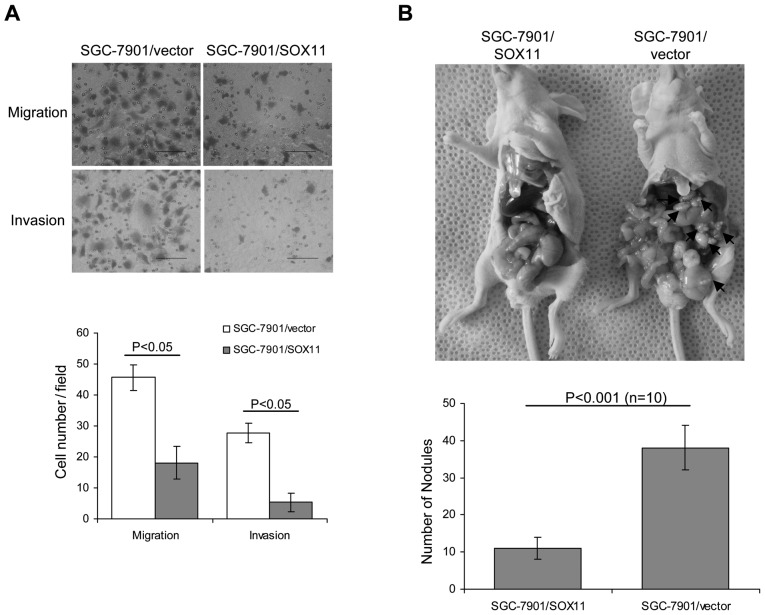
SOX11 overexpression suppressed gastric cancer cell metastasis *in vitro* and *in vivo*. (A) SOX11 overexpression suppressed SGC-7901 cell migration and invasion. Representative images of migrated/invaded cells are shown (left, original magnification, ×200). Number of migrated/invaded cells is plotted (right). (B) Effects of SOX11 overexpression on suppressing peritoneal spreading and metastasis. Metastatic nodules are obvious in the control group. The numbers (mean ± SD) of metastatic nodules in control and SOX11-overexpression group are plotted (n=10).

We next asked whether overexpression of SOX11 affected gastric cancer migration and invasion. Boydon chamber assays were used to investigate the *in vitro* ability of migration and invasion in SOX11-overexpression and vector-control cells. As expected, SOX11 overexpression suppressed cell migration and invasion (Fig. 4A).

As peritoneal spreading and metastasis are common in gastric cancer and are pivotal factors for its poor prognosis, we used a nude mouse model to investigate the influence of SOX11 levels on peritoneal metastasis. Consistent with *in vitro* observations, we found that SOX11-overexpressing cells formed less metastatic nodules than vector-control cells (Fig. 4B). Our data suggest that SOX11 overexpression suppresses cell migration and invasion.

### SOX11 protein expression in gastric cancer tissues and non-tumor tissues

As *in vitro* and *in vivo* cell model analyses strongly suggested a tumor-suppressor role of SOX11 in gastric cancer cells, we asked whether the expression pattern of SOX11 human gastric cancer tissues agreed with the cell study. We performed immunohistochemistry staining to examine the SOX11 protein expression in gastric cancer tissues. Gastric tumor and paired non-tumor tissues from 151 patients were stained. Of the gastric cancer tissues 51.7% (78 of 151) showed positive SOX11 staining (IHC score: 4–12), and 48.3% (73 of 151) of the samples showed negative staining (IHC score: 0–3). A significant correlation was found between positive expression of SOX11 and TNM stage (I+II, P=0.031), Lauren’s classification (intestinal type, P<0.001) and differentiation status (high and medium, P<0.001) (Table I). Of note, the SOX11 positive percentile was higher in lymph node metastasis negative and stage (T1+T2) tumors than lymph node metastasis positive and stage (T3+T4) tumors, although this was not statistically significant (Table I). These data suggest that SOX11 is highly expressed in a subset of human gastric cancer, which shows less malignant characteristics according to clinicopathological features.

### SOX11 expression is an independent prognostic factor and associated with better survival in gastric cancer patients

**Figure 5.**
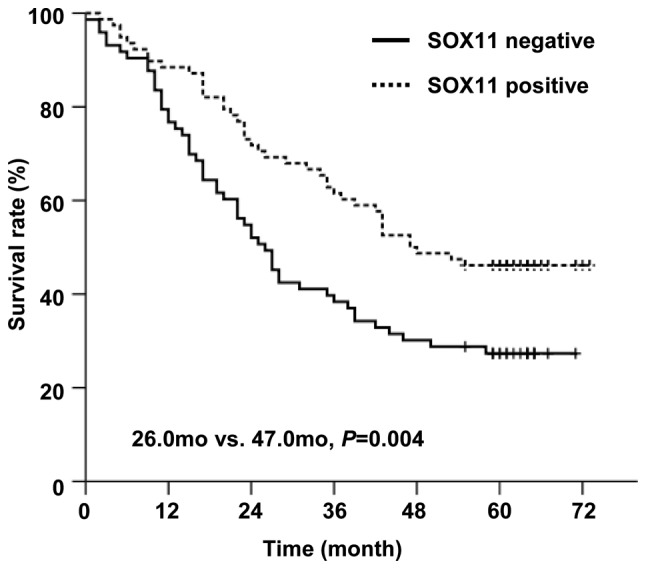
Survival analysis of SOX11 in gastric cancer. Kaplan-Meier analysis and the log-rank test identified SOX11 as significantly associated with cancer specific survival in high grade tumors.

SOX11 was recently discovered to be a prognostic factor in lymphoma and ovarian cancer (11,20). To investigate the prognostic significance of SOX11 in gastric cancer, we analyzed the correlation of SOX11 with survival of patients using Kaplan-Meier analysis. We discovered that patients in the SOX11-positive group showed longer overall survival than those in SOX11-negative group (medium survival: 47.0 months vs. 26.0 months, P= 0.004, Fig. 5). Our data suggested an association between SOX11 expression and improved overall survival in gastric cancer patients.

**Figure 6.**
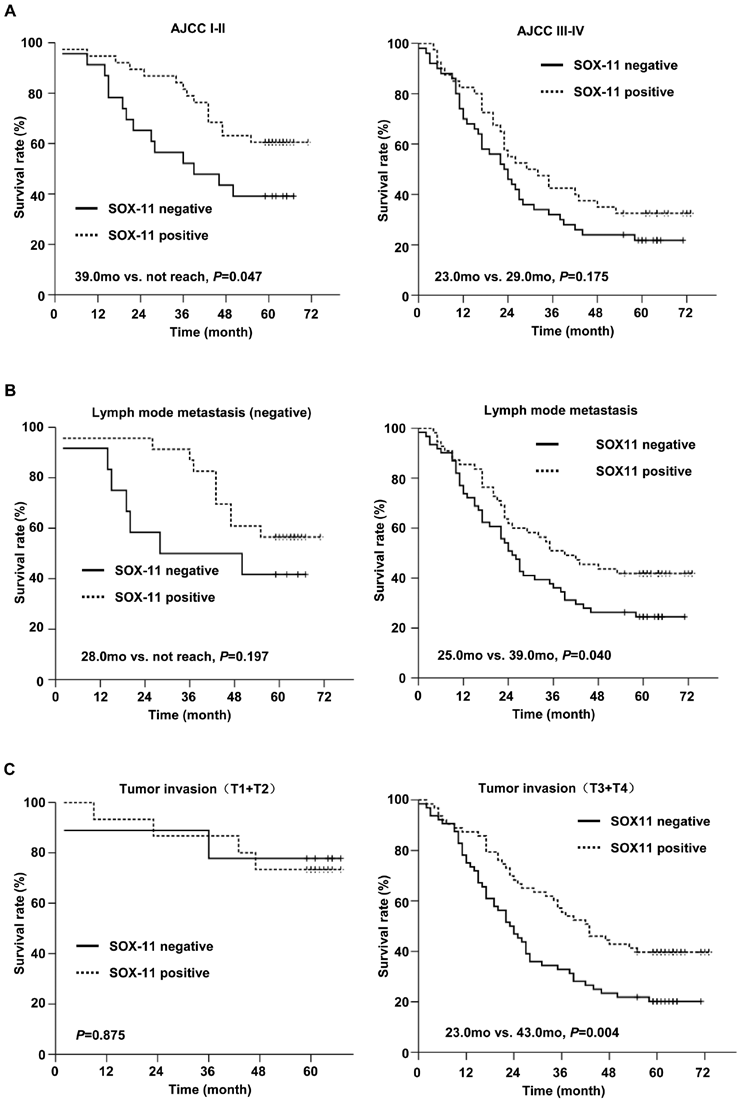
Survival analysis of SOX11 in gastric cancer stratified by stage. (A) Kaplan-Meier survival curves in gastric patients stratified by AJCC staging. (B) Kaplan-Meier survival curves in gastric patients stratified by N stage. (C) Kaplan-Meier survival curves in gastric patients stratified by T stage. The patients were classified into two groups based on SOX11 expression.

Cases with negative lymph node status and early tumor invasion stage tended to have a higher SOX11 positive rate. We stratified the patients with TNM stage, and further stratified by N and T stage to evaluate the prognostic value of SOX11. In 61 patients whose TNM stage was I and II, SOX11-positive group had longer survival than SOX11-negative group (P=0.047, Fig. 6A, right). In patients whose TNM stage was III and IV, SOX11-positive group showed a trend of longer survival than SOX11-negative group, but not statistical significance (Fig. 6A, left). In 116 patients with lymph node metastasis, 55 cases (47.4%) were SOX11-positive and 61 cases (52.6%) were SOX11-negative. Kaplan-Meier analysis showed that patients in SOX11-positive group had improved survival compared to SOX11-negative group (medium survival: 39.0 months vs. 25.0 months, P=0.04, Fig. 6B, right). Similarly, in a group of 127 patients at late tumor invasion stage (T3+T4), 63 cases (49.6%) of the SOX11-positive group showed longer medium survival (43.0 months) than the other 64 cases (50.4%) of the SOX11-negative group (23.0 months) patients (P=0.004, Fig. 6C, right). SOX11 was not significantly correlated with survival in patients without lymph node metastasis (Fig. 6B, left) and at early tumor invasion stage (T1+T2) (Fig. 6C, left). These data suggest that the expression of SOX11 is a prognostic factor for improved survival in patients with lymph node metastasis and at advanced tumor invasion stage.

Further multivariate analysis showed that tumor invasion (relative risk = 2.155, 95% CI, 1.531–3.032, P<0.001), lymph node metastasis (relative risk = 1.345, 95% CI, 1.099–1.647, P=0.004), SOX11 (relative risk = 0.604, 95% CI, 0.391–0.9340, P=0.023), and age (relative risk = 1.913, 95% CI, 1.213–3.019, P=0.005) were independent prognostic factors for the survival rate of gastric cancer patients (Table II). These data demonstrate that SOX11 expression is an independent positive prognostic factor in gastric cancer.

## Discussion

SOX11 exhibits a wide and highly dynamic expression during embryogenesis (9). SOX11 has been previously studied in lymphoma and ovarian cancers and showed correlations to patients’ survival. We investigated the role of SOX11 overexpression on gastric cancer malignant behavior. SOX11 overexpression suppressed migration and invasion ability of gastric cancer cells *in vitro* and *in vivo*. We further examined the expression and clinical significance of SOX11 in gastric cancer. We uncovered SOX11 as a prognostic factor for improved survival in gastric cancer patients.

SOX11-deficient mice exhibited severe defects in several organ systems which all express SOX11 at times of extensive remodeling. SOX11-deficience causes stomach hypoplasia in embryos (9). However, the function of SOX11 in gastric cancer is unclear. From the Oncomine database, we found that SOX11 was overexpressed in intestinal-type gastric cancer. This finding suggested that overexpressin of SOX11 might be intestinal-type related and suggest a favorable outcome. SOX11-overexpressing gastric cancer cells showed suppressed migration and invasion malignant behavior. In embryogenesis, SOX11 expression accompanies the activation of several signal transduction pathways, including Wnt, transforming growth factor β (TGF-β), bone morphogenetic protein (BMP), fibroblast growth factor (FGF) and Hedgehog signaling (21). However, the role of SOX11 in tumorigenesis is poorly understood. In hematopoietic malignancies, SOX11 knockdown and overexpression were found to be correlated with increased and decreased cell proliferation, respectively (10). Rb-E2F growth regulatory pathway was involved in this growth regulatory role of SOX11 by signaling pathway analysis (10). However, we showed that SOX11 levels did not affect gastric cancer cell growth. The cell cycle and apoptosis analysis also showed no difference between SOX11-overexpressing and vector-control cells. These results suggested that SOX11 did not suppress gastric tumorigenesis through regulating cell growth. We further discovered that SOX11 overexpression strongly inhibited *in vitro* cell migration and invasion as well as *in vivo* peritoneal metastasis. SOX11 overexpression may inhibit gastric tumorigenesis through suppressing cancer cell motility.

SOX11 has been studied in various lymphoproliferative diseases. However, the prognostic relevance of SOX11 remains unclear since it is found to be associated with both improved and reduced survival. Consistent with previous finding which showed SOX11 as a tumor-suppressor in ovarian cancer (20), we found SOX11 was correlated with less malignant features in gastric cancer. Specifically, SOX11 is expressed in early stage, differentiated and intestinal-type gastric cancer. Furthermore, SOX11 expression was correlated with improved survival in gastric cancer patients. Cox regression multivariate analysis also reveals that SOX11 is an independent prognostic factor for predicting survival of patients. It is noteworthy that SOX11 is a correlated with longer survival in patients with lymph node metastasis and deep tumor invasion, suggesting that SOX11 is a predictor of patient survival even in advanced stage. As gastric cancer patients are often diagnosed at advanced stage, the prognostic value of SOX11 in advanced stage patients could be of great importance in predicting patient survival. These findings were also consistent with the results from SOX11-overexpression cell models, which suggested a tumor-suppressor role of SOX11 in gastric cancer. Together, our study reveals the correlation of SOX11 with clinicopathological features and survival of patients.

In concusion, our findings demonstrate that SOX11 suppresses gastric cancer migration and invasion *in vitro* and *in vivo*. SOX11 may serve as a marker for a subgroup of gastric cancer which shows less aggressive features and better prognosis.

## Figures and Tables

**Table I.** Clinicopathological characteristics of SOX11 in gastric cancer.

	SOX11		
Clinicopathological characteristics	Negative (%)	Positive (%)	P-value
Gender			
Male	54 (51.9)	50 (48.1)	0.191
Female	19 (40.4)	28 (59.6)	
Age			
≤60	29 (55.8)	23 (44.2)	0.186
>60	44 (44.4)	55 (55.6)	
Tumor invasion			
T1–T2	9 (37.5)	15 (62.5)	0.246
T3–T4	64 (50.4)	63 (49.6)	
Lymph node metastasis			
Negative	12 (34.3)	23 (65.7)	0.058
Positive	61 (52.6)	55 (47.4)	
Distant metastasis			
Negative	70 (49.6)	71 (50.4)	0.382
Positive	3 (30.0)	7 (70.0)	
TNM staging			
I–II	23 (37.7)	38 (62.3)	0.031
III–IV	50 (55.6)	40 (44.4)	
Lauren’s type			
Intestinal	27 (29.3)	65 (70.7)	<0.001
Diffuse	46 (78.0)	13 (22.0)	
Differentiation			
High	6 (22.2)	21 (77.8)	<0.001
Median	13 (25.0)	39 (75.0)	
Low	54 (75.0)	18 (25.0)	

**Table II.** Multivariate Cox regression analysis of the association of SOX11 expression with gastric cancer patient survival.

Parameter	HR (95% CI)	P-value
Tumor invasion	2.155 (1.531–3.032)	<0.001
Lymph node metastasis	1.345 (1.099–1.647)	0.004
SOX11	0.604 (0.391–0.934)	0.023
Age	1.913 (1.213–3.019)	0.005
